# Electrically Driven Site-Controlled Single Photon Source

**DOI:** 10.1021/acsphotonics.3c00097

**Published:** 2023-07-05

**Authors:** Shi Guo, Savvas Germanis, Takashi Taniguchi, Kenji Watanabe, Freddie Withers, Isaac J. Luxmoore

**Affiliations:** † Department of Physics and Astronomy, University of Exeter, Exeter EX4 4QL, United Kingdom; ‡ Department of Engineering, University of Exeter, Exeter EX4 4QF, United Kingdom; § International Center for Materials Nanoarchitectonics, National Institute for Materials Science, 1-1 Namiki, Tsukuba 305-0044, Japan; ∥ Research Center for Functional Materials, National Institute for Materials Science, 1-1 Namiki, Tsukuba 305-0044, Japan

**Keywords:** single-photon source, site-control, electroluminescence, quantum confined Stark effect, WSe_2_

## Abstract

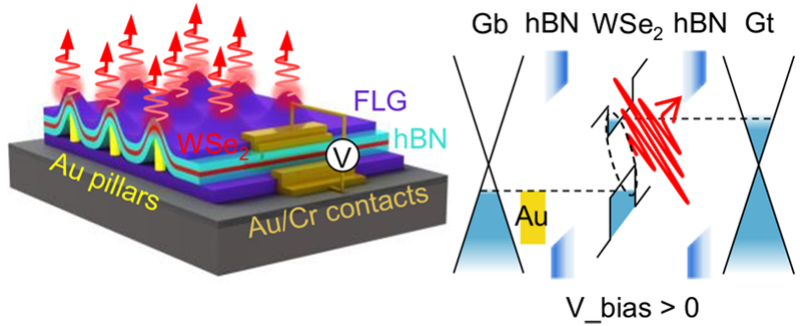

Single photon sources are fundamental building blocks for quantum communication and computing technologies. In this work, we present a device geometry consisting of gold pillars embedded in a van der Waals heterostructure of graphene, hexagonal boron nitride, and tungsten diselenide. The gold pillars serve to both generate strain and inject charge carriers, allowing us to simultaneously demonstrate the positional control and electrical pumping of a single photon emitter. Moreover, increasing the thickness of the hexagonal boron nitride tunnel barriers restricts electroluminescence but enables electrical control of the emission energy of the site-controlled single photon emitters, with measured energy shifts reaching 40 meV.

## Introduction

The goal of developing a commercially viable solid-state single photon source (SPS) has been the focus of an extensive research effort for several decades and many material systems have been considered.^1^ The design requirements for commercially practical devices are extensive and include on-demand operation, high brightness, and routes to scaleable manufacturing. The current state of the art is based on III–V quantum dots (QD), where high brightness and high photon indistinguishability have been simultaneously demonstrated.^2,3^ Defect centers in wide-bandgap semiconductors, such as diamond^4−7^ and SiC,^8^ are another area of considerable progress. In particular, negatively charged nitrogen vacancies in diamond are a proven source of single^4^ and indistinguishable^5^ photons, but are limited by the low fraction of light emitted into the zero phonon line (ZPL). Other defects, such as negatively charged silicon vacancies offer better performance,^9^ but the diamond host provides a considerable challenge to scalable fabrication and photonic integration.

In general, two of the major challenges in the development of solid state single photon sources are the deterministic fabrication of the underlying single photon emitter and achieving electrically driven operation. The vast majority of single photon sources are optically pumped, whereas an electrically pumped device brings obvious benefits in terms of miniaturization, efficiency, and scalability. To date, only a handful of electrically pumped single photon sources have been demonstrated, based on self-assembled^3^ and colloidal QDs,^10^ defect centers in diamond^7^ and SiC,^8^ and single molecules.^11^

The challenges of deterministic fabrication and electrical pumping can be addressed with two-dimensional materials. Mono and bilayers of transition metal dichalcogenides (TMDCs), in particular WSe_2_, can host single photon emitting defects,^12−16^ at around 750 to 800 nm, a wavelength range that is compatible with silicon based avalanche photo diodes (APD), silicon nitride integrated photonics,^17^ and proposed satellite-based quantum communication windows.^18^ Furthermore, emitters can be deterministically induced^19,20^ and integrated with nanophotonics^21,22^ via strain engineering and incorporated in heterostructure devices that enable electrical tuning^23−25^ and injection.^26−29^

In this Letter, we report a vertical tunneling light emitting diode that incorporates strain engineering within a van der Waals heterostructure and thereby enables electrically pumped single photon emission from a site-controlled defect within a WSe_2_ monolayer. Furthermore, by controlling the thickness of the tunnelling barriers, the electroluminsence (EL) can be suppressed in favor of gate-tuneable photoluminescence (PL) emission with a tuning range of ∼40 meV.

## Results and Discussion

**Figure 1:**
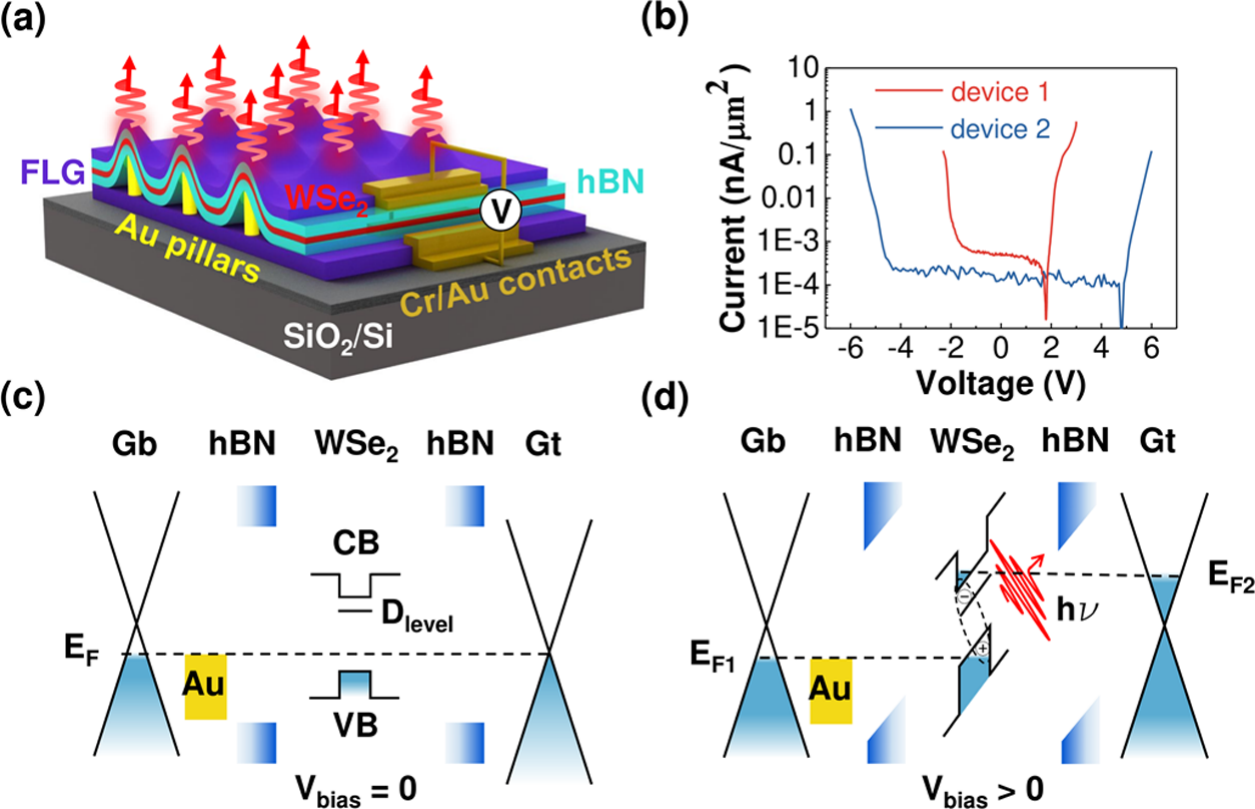
Design and operation of the WSe_2_ vertical tunneling light emitting diode. (a) Schematic diagram of the device structure. From top to bottom: few-layer graphene (FLG)/hBN/WSe_2_/hBN/gold pillars/FLG. The gold pillars are fabricated on top of the bottom FLG. The bias voltage is applied between the top and bottom FLG electrodes. (b) *I*–*V* characteristic of device 1 (2) with thin (thick) hBN barriers. (c, d) Energy band diagram of the device at pillar sites with and without applied bias, respectively. The presence of the gold pillar pins the Fermi level of the bottom FLG. Under finite bias, the band bending of WSe_2_ and the tuning of the Fermi level (E_F2_) of the top FLG lead to the tunneling of electrons and holes into WSe_2_.

The device geometry is illustrated schematically in Figure 1a. To fabricate the device, a few-layer graphene (FLG) flake is exfoliated onto a Si substrate covered with a 300 nm thick SiO_2_ layer. Then an array of gold pillars with a pitch of 5 μm is fabricated on top of the graphene flake by using electron beam lithography and thermal evaporation. Based on previous reports,^19,30^ we select the height and diameter to be 100 and 300 nm, respectively, to give a pillar aspect ratio of ∼0.3. This is to ensure that the strain in the WSe_2_ layer is sufficiently large to give a high probability of inducing single photon emitters at the pillar sites. This is confirmed by the PL maps of the two devices presented in this work where bright areas, corresponding to narrow emission lines, are observed at all pillar sites (see Figures 4b and SI, Figure S2). Next, we use the “all-dry transfer method”^31^ to stack hBN, monolayer WSe_2_, hBN, and FLG in sequence on top of the gold pillars/FLG structure. Lastly, chromium/gold (5 nm/50 nm) contacts are made to the top and bottom FLG layers, which act as transparent electrodes. The role of the gold pillars is 2-fold. First, to generate strain and induce localized single-photon emitters, as more commonly achieved with dielectric pillars.^16,20,32,33^ And second, the pillars provide a channel for charge carrier injection at the high strain regions.

**Figure 2:**
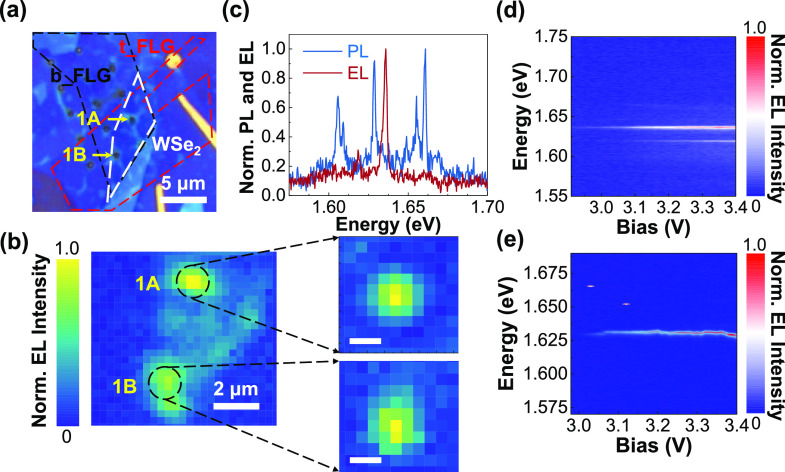
Electrically driven site-controlled single photon emitters. (a) Microscope image of device 1. t_FLG and b_FLG denote top and bottom few-layer graphene respectively. Two pillars are included in the active region which has the same geometry as in Figure 1a. (b) EL map from the whole active area indicated in (a), for a bias voltage of 3.2 V, illustrating that the emission is mainly from the apex of the pillars 1A and 1B. The two closeup images are high resolution EL maps on pillar 1A and pillar 1B, respectively, with the integrated energy ranging from 1.55 to 1.65 eV, showing that the EL is localized to the pillar sites. The scale bar is 1 μm. (c) PL and EL spectra taken from pillar 1A. PL is taken at bias = 0 V and EL is taken with bias = 3.2 V. (d) and (e) are the EL spectra from pillars 1A and 1B, respectively, as a function of bias voltage.

The band structure of the device is sketched in Figure 1c,d, without and with an external bias voltage, respectively. The presence of gold pillars modulates the energy band landscape of WSe_2_, leading to a minima in the conduction band. The mixing of deep defect-related energy levels in the bandgap^34^ and the strain-induced conduction band minima leads to local lattice symmetry breaking, thus enabling efficient intervalley transitions for dark excitons and bright luminescent emission.^30,35^ In our device, the neutral excitons are created by direct electrical doping from the bottom gold pillars and top FLG. At zero bias, the Fermi level of the whole system remains in equilibrium, lying within the bandgap of the monolayer WSe_2_, as shown in Figure 1c, where the Fermi level of the bottom FLG is pinned by the gold pillars. Under finite forward bias, the quasi-Fermi level (E_F2_) of the top FLG can be tuned upward, resulting in tunneling of electrons through the hBN barriers into the monolayer WSe_2_. Meanwhile, the valence band of WSe_2_ can also bend above the Fermi level of gold, resulting in the injection of holes (Figure 1d). The injected charge carriers are trapped in the strain-defect mixed states and recombine radiatively, thus, producing bright single photon emission.

Here we present two devices with different hBN barrier thickness. In device 1, the top and bottom hBN barriers are 3–5 monolayers thick (see SI, Figure S2), which is favorable for efficient charge carrier tunnelling, and therefore, electrically driven emission.^36^ In contrast, device 2 consists of hBN barriers that are >10 layers thick (see SI, Figure S4), which prevents tunneling and instead enables electric-field tuning of the single photon emission under optical excitation. Figure 1b presents the current–voltage (*I*–*V*) characteristics of the two devices. Both devices show typical back-to-back Schottky-diode characteristics with slight asymmetry of the forward and reverse turn-on voltages. This is most likely due to small differences in the thickness of the top and bottom hBN barriers, as well as asymmetry in the top and bottom electrode work functions due to differences in doping and the presence of the gold pillars at the bottom electrodes. It has been shown that the tunnelling current decreases exponentially for increased hBN barrier thickness^37^ and this is reflected in the *I*–*V* curves of the two devices: the tunnelling current density increases rapidly at smaller bias for thin hBN barriers; whereas, the tunnelling current density only increases exponentially for thick hBN barriers when |*V*| > 5 V.

We first present electrically pumped single photon emission from device 1, which has thin hBN barriers. Figure 2a presents an optical image of this device, where the active region is indicated by white dashed lines. There are two pillars within this active region, which we label as 1A and 1B. Spatially controlled electroluminescence is demonstrated by the EL map, shown in Figure 2b, where two maxima of the EL are observed, corresponding to the position of the two pillars. The emission intensity from the rest of the active region is relatively weak and is attributed to emission from shallow defect-related localized excitons and delocalized neutral excitons^38,39^ (see SI, Figure S1).

High resolution EL maps on both pillar sites are also presented in Figure 2b (the closeups), with the energy selected from 1.55 to 1.65 eV. The EL emission is clearly localized to the pillar sites, and the spacial profile of EL intensity also matches well with the spacial profile of the pillars (SI and Figure 2c,d). The EL intensity reaches its maximum at the pillar apex, which has the maximum strain and decreases in the surrounding regions, which reflects a decreasing strain effect.^19,30^

**Figure 3:**
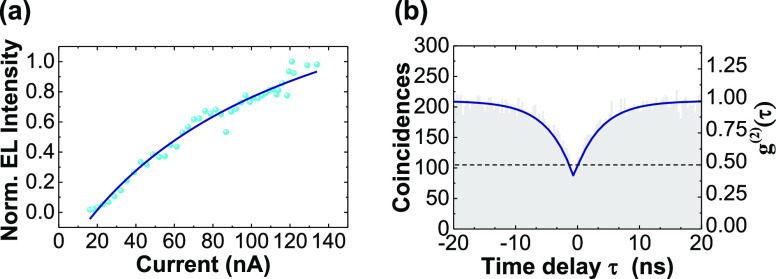
Single photon nature of the emitter from pillar 1B. (a) Normalized integrated EL intensity recorded at the pillar 1B as a function of tunnelling current. The blue line is the fit to the data of EL = EL_max_ × ((*I* – *I*_0_)/(*I* – *I*_0_ + *I*_sat_)), where EL denotes the integrated intensity of electroluminescence and EL_max_ is the maximum EL intensity. *I*, *I*_0_, and *I*_sat_ is the current through the device, the current at which EL starts to emerge, and the current at which EL starts to saturate, respectively. (b) Photon correlation function, *g*^2^(τ) from pillar 1B under the electrical drive. The blue line shows a fit to the data, which reveals *g*^(2)^(0) = 0.32 ± 0.01.

Figure 2d and e demonstrate the bias-dependent EL spectra of pillar 1A and pillar 1B, respectively. Both pillar sites show sharp peaks, which we attribute to strain-induced SPE. At pillar 1A, the EL spectrum consists of two peaks at around 1.62 and 1.63 eV, respectively, as shown in Figure 2c, which originate from either two separate emitters or different charge states of a single emitter. The PL spectra at zero bias is plotted for comparison. The main emission line is blue-shifted by ∼8 meV and there are additional PL lines. These differences can be attributed to a Stark Shift (see SI and Figure 3a) and photoexcitation of additional charge states and nearby emitters, respectively. The emission energy is stable over the measured bias range, indicating reduced spectral wandering compared with pillar 1B (Figure 2e). At pillar 1B, the EL spectrum is dominated by a sharp peak at around 1.63 eV. From the bias-dependent EL spectra plotted in Figure 2e, it can be seen that the turn-on voltage of the single photon EL is approximately 3 V. With increasing bias, the intensity increases and there is a small amount of spectral wandering in the emission energy. The integrated EL intensity as a function of the current through the diode is plotted for pillar 1A (SI, Figure S3) and pillar 1B (Figure 3a), respectively, and exhibit the saturation behavior expected for a single-photon emitter.^26^

To confirm the single photon nature of the EL emission, we measure the second order correlation function, *g*^(2)^(τ). Presented in Figure 3b is *g*^(2)^(τ) of the EL emission from pillar 1B under a bias voltage of 3.2 V. To account for the ∼15 nm bandwidth of the detection filter, the data is background corrected following ref (40). A fit to the data yields *g*^(2)^(0) = 0.32 (without background correction, *g*^(2)^(0) = 0.46), below the 0.5 threshold for a single photon emitter. This result confirms that with this device architecture we can realize electrically driven single photon emission from a site-controlled location. From the same fit, we extract the radiative lifetime of the emitter, τ_r_ ≈ 3.0 ns, which is typical for single photon emitters in WSe_2_,^13,14,16^ and suggests there is no plasmonic enhancement of the spontaneous emission.^21,22^ This is not unexpected as the dimensions of the pillar are such that its plasmonic resonance would lie at much longer wavelength than the single photon emitter.^41,42^ It was not possible to confirm the single photon nature of the emission from pillar 1A due to irreversible breakdown of the diode during high bias voltage measurements of pillar 1B. However, given the similarity of the spectra (Figure 2d,e and the saturation behavior (Figure 3a and SI, Figure S3b) of the two pillars, it is reasonable to expect, given a sufficiently narrow filter is used, that pillar 1A would also have yielded *g*^(2)^(0) < 0.5.

**Figure 4:**
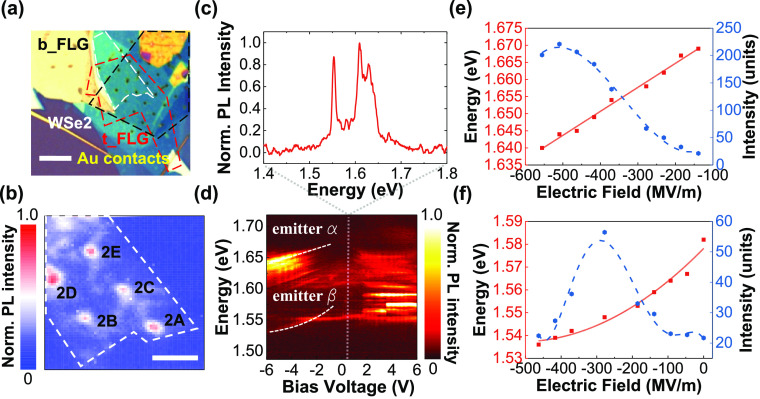
Charge tunable single photon emitters. (a) Microscopic image of device 2. There are five pillars inside the active region. The scale bar is 5 μm. (b) Raster scan of the integrated PL intensity over the active region of device 2. The spectral integrated range is from 1.5 to 1.7 eV. The scale bar is 5 μm. (c) The PL spectrum of pillar 2B taken at zero bias voltage. (d) Voltage dependent PL spectra from pillar 2B, showing the Stark shift of several emitters under applied electric fields. (e, f) Emission energy and intensity change of emitter-α and emitter-β as a function of electric fields, respectively. Red solid lines are the fitting to the data by eq 1 and blue dashed lines are guides to the eye, showing the change trend of PL intensity.

We now turn our attention to device 2, pictured in Figure 4a, which has much thicker hBN barriers. Both top and bottom hBN barriers are about 10 layers thick, which is sufficient to suppress EL for bias voltages in the range ±6 V. A map of the photoluminescence from the active region of device 2 is shown in Figure 4b. There are 5 pillars within the active region, with enhanced PL emission recorded from all of the pillar sites. Figure 4c shows the PL spectra from pillar 2B at a zero bias voltage. Similar spectra are also observed from the other four pillars, with narrow emission peaks from localized excitons in the range from 1.55 to 1.65 eV (see SI, Figure S5).

To further study the effect of external electric fields on the localized excitons, PL spectra are recorded as a function of bias voltage, with an example shown in Figure 4d. For voltages larger than ∼±1.5 V extra emission peaks emerge, due to the fact that nonradiative recombination is suppressed under higher bias. From these spectra, we choose two emitters to study in further detail, labeled α and β in Figure 4d (further analysis of other emitters is presented in the SI, Figures S6–S9). Close to zero bias, emitter-α is located at about 1.64 eV and demonstrates a linear Stark shift of ∼30 meV (Figure 4e). The PL emission intensity first increases before decreasing with increasing electric fields. This behavior is likely due to the suppression of nonradiative recombination at moderate electric fields, followed by separation of the electron–hole pair and reduced radiative recombination at strong electric fields. The large energy shift and intensity change are the hallmark of the quantum confined Stark effect. A similar energy shift is also observed for emitter-β (Figure 4f), but with a quadratic Stark shift and a maximum tuning range of ∼40 meV. Compared with the energy shift of 1.1 and 1.8 meV for neutral excitons and trions, respectively,^25^ these PL emission peaks can be attributed to localized excitons which are induced by the interplay between strain and defects.^23,43^

The change of dipole moment and polarizability are then extracted from a fit of the data to the equation:
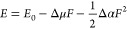
1where *E* is the emission energy, *E*_0_ is the zero-field transition energy, and Δμ and Δα denote the change of dipole moment and polarizability between the excited and ground states, respectively. *F* is the local electric field applied to the emitter and is approximated as , where *V* is the external bias, ϵ is the dielectric constant of the encapsulating hBN layers and *t* is the thickness of device.^23,44^ From AFM measurements (see SI, Figure S4) device 2 is ∼18 nm thick and the dielectric constant of hBN is taken to be 3.^45^

The change of dipole moment of emitter-α is calculated to be 3.35 D (1 D = 3.33 × 10^–30^C·m), as shown in Figure 4e. Emitter-β has a larger dipole moment change of 6.35 D and a negative polarizability volume of −442 Å, as in Figure 4f. Negative polarizability is likely to result from the reduced direct and exchange Coulomb interaction of localized excitons under electric fields.^43^ However, the quadratic Stark effect is not prevalent in our geometry and most emitters from all five pillars show a linear Stark shift, all with a tuning range of 20–30 meV (see SI, Figures S6–S9). A similar emitter, which we label as emitter-α at ∼1.64 eV, is found in all five pillars, and we find the change of dipole moment is in the range of 3 ± 0.5 D, and the energy change per unit bias is in the range of 5 ± 0.5 meV/V (SI, Figure S10). Both values match well with theoretical calculations and previous reports, but with less variations.^23,35^ The predominant linear Stark shift is also an indicator that these localized excitons, created by pillar-induced strain and defects, have a noncentrosymmetric atomic configuration.^44^ Under reverse bias, there is some evidence for charge state switching^46^ and further work is required to better understand the microscopic origin of emitter tuning and the role played by local charge variations across the WSe_2_ and FLG layers, particularly in the presence of the metal pillars. For example, this could be investigated with devices that include independent electrical gating of the WSe_2_ carrier density.

Stark tuning is observed in device 1, below the turn-on voltage (see SI, Figure S3a), which is not unexpected given the similarity of the two devices. On the other hand, it was not possible to observe EL from device 2. Increasing the voltage beyond 6 V did not yield a measurable signal and ultimately lead to breakdown of the device. We attribute this to the exponential dependence of tunnelling current with increasing barrier thickness^36,37^ versus the linear dependence of the breakdown voltage, and which highlights the importance of thin hBN barriers in this device architecture.

## Conclusion

In summary, our work presents a novel device architecture that integrates metallic pillars into a 2D-material heterostructure diode, thus realizing a site controlled, electrically driven single photon source. Through control of the hBN barrier thickness, we show that the device modality can be changed to enable electrical control of the emission energy under optical pumping, with Stark shifts up to 40 meV measured and consistent behavior across multiple emitters. These results represent an important step for the integration of TMDC-based single photon devices into quantum photonic technologies. The device architecture, where the emitter localization and electrical injection are realized through standard lithographic techniques means it is compatible with scaleable semiconductor device fabrication and could in-future be combined with wafer-scale growth of 2D materials.^47^ The architecture can be optimized so that the metallic pillar also acts as a plasmonic antenna, to enhance the spontaneous emission rate and the directional efficiency^21,22^ of the device, and is compatible with further integration, such as the on-chip routing^48^ and readout^49^ of single photons.

## Experimental Methods

### Device Fabrication

FLG flakes and hBN layers are first exfoliated onto 300 nm SiO_2_/Si substrates. Monolayer WSe_2_ is exfoliated on a polydimethylsiloxane (PDMS) sheet (SI, Figure S1). The FLG and hBN flakes of the required thickness are identified by their contrast in an optical microscope. Monolayer WSe_2_ is identified by PL measurement as shown in SI, Figure S5f. About 100 nm high gold pillars, with a 5 nm chromium adhesion layer are fabricated using electron beam lithography and thermal evaporation. A home-built transfer setup is used to fabricate the heterostructure. The stamp consisting of PDMS and polypropylene carbonate (PPC) is used to pick up and transfer the flakes following the method reported in ref (31).

### Optical Measurements

The optical experiments are performed at 5 K in a home-built microluminescence system equipped with a closed-cycle cryostat. Photoluminescence is excited using a 633 nm CW diode laser coupled to a long working distance microscope objective (N.A. = 0.8), mounted on an XYZ piezo stage. The luminescence from the sample is collected using the same objective and directed, via a single-mode (core diameter, ⌀ = 4.4 μm or multimode (⌀ = 50 μm) fiber to a monochromator/CCD for spectroscopy, or to a Hanbury-Brown Twiss (HBT) interferometer. The HBT setup consists of a single mode fiber beam splitter coupled to a pair of avalanche photo diodes (APD) and a time-correlated single photon counting module. The luminescence is filtered using a tunable band-pass filter (bandwidth ∼ 15 nm). The fiber also acts as a spatial filter and restricts light collection to an area of the sample determined by the core diameter of the fiber. Device 1 (2) measurements in the main text are recorded with single-mode (multimode) fiber. As a result, more emission lines are observed in the spectra from the pillar sites of device 2 compared to those in device 1. See also SI, Figure S3a, which presents PL spectra from device 1 recorded with a multimode fiber. A source-measurement unit is used to apply the voltage and measure the current passing through the device.

## Supporting Information Available

Optical and topographical characterization data for devices 1 and 2; Method of second order correlation background correction; Further low temperature characterization of devices 1 and 2 (PDF)
