# Supplementary material for: Electrically Driven Site-Controlled Single Photon Source
Source: ACS Photonics. 2023 Jul 5;10(8):2549–55. doi: 10.1021/acsphotonics.3c00097 (PMC10436352; doi:10.1021/acsphotonics.3c00097)
Supplement: Supplementary file 1 — ph3c00097_si_001.pdf [file ph3c00097_si_001.pdf]

Supporting information: Electrically Driven Site-Controlled Single Photon Source

Shi Guo,[†] Savvas Germanis,[‡] Takashi Taniguchi,[¶] Kenji Watanabe,[§] Freddie Withers,[†] and Isaac J. Luxmoore[*,‡]

†*Department of Physics and Astronomy, University of Exeter, EX4 4QL, United Kingdom*

‡*Department of Engineering, University of Exeter, EX4 4QF, United Kingdom*

¶*International Center for Materials Nanoarchitectonics, National Institute for Materials Science, 1-1 Namiki, Tsukuba 305-0044, Japan*

§*Research Center for Functional Materials, National Institute for Materials Science, 1-1 Namiki, Tsukuba 305-0044, Japan*

E-mail: i.j.luxmoore@exeter.ac.uk

Contents

Optical and topographic characterization of the devices **3**

Second order correlation function with background subtraction correction **4**

List of Figures

S1 Optical characterization of monolayer WSe_2. 5

S2 Optical and AFM characterization of device 1 5

S3 Electric field tuning and electroluminescence saturation of pillar 1A. 6

S4 SEM and AFM characterization of device 2. 6

S5 PL spectra at different pillars of device 2. 7

S6 Bias dependent PL emission spectra of pillar 2A. 7

S7 Bias dependent PL emission spectra of pillar 2C. 8

S8 Bias dependent PL emission spectra of pillar 2D. 8

S9 Bias dependent PL emission spectra of pillar 2E. 9

S10 Change of dipole moment and energy change per unit bias of all 5 pillars. . . 9

Optical and topographic characterization of the devices

Fig. S1a is a typical optical image of WSe_2 flakes exfoliated on the PDMS sheet. The monolayer is selected using the optical contrast and verified by observing the neutral delocalised exciton PL emission at about 1.73 eV and shallow defect-related localised exciton emission centered at 1.65 eV[1–3] (Fig. S5f). Fig. S1b is the electroluminescence (EL) of WSe_2, taken at a flat region, away from the pillars, of device 1. The EL spectrum is consistent with the monolayer PL spectrum.

The PL mapping of device 1 is shown in in Fig. S2a and the AFM measurement is shown in Fig. S2b. The high resolution of AFM spacial profiles on the pillar sites in Fig. S2c and d confirms that the spacial profile of the pillars is the same as the EL intensity enhancement profile in the EL and PL map, which is a evidence that pillar induced strain can result in site-controlled EL and PL single photon emission. The thickness of device 1 is about 6 nm in total and the height of the pillars is about 100 nm (Fig. S2e and Fig. S2f).

Fig. S4 shows the AFM and SEM measurements of device 2. Fig. S4a and d confirm the pillar diameters (300 nm) and the total thickness (18 nm) of device 2 respectively. From Fig. S4c, we can confirm the height of the pillars to be $\sim$ 90 nm. Fig. S5 is the PL spectra of the rest of pillars. Compared with the broad shadow-defect emission, there are multiple discrete emission lines at the energy below 1.65 eV.

Fig. S6 to Fig. S9 are the bias dependent PL spectra of the rest pillars in device 2 and Fig. S10 is the summary of the extracted dipole moment and the energy change per unit bias of all five pillars.

Second order correlation function with background subtraction correction

The second order correlation function $g^{(2)}(\tau)$ is calculated in this paper by the equation:[4]

$$g^{(2)}(\tau) = C_N(\tau)\frac{S_1S_2 + 2S_1B_2 + 2S_2B_1 + B_1B_2}{S_1S_2} - \frac{2S_1B_2 + 2S_2B_1 + B_1B_2}{S_1S_2} \tag{1}$$

where $C_N(\tau)$ is the normalised coincidence amplitude, B_1 and B_2 are the unbalanced dark-count rates on both two detectors, S_1 and S_2 are the signal counts. Before the background emission subtraction, $g^{(2)}(0)$ gives the value of 0.46 ± 0.01.

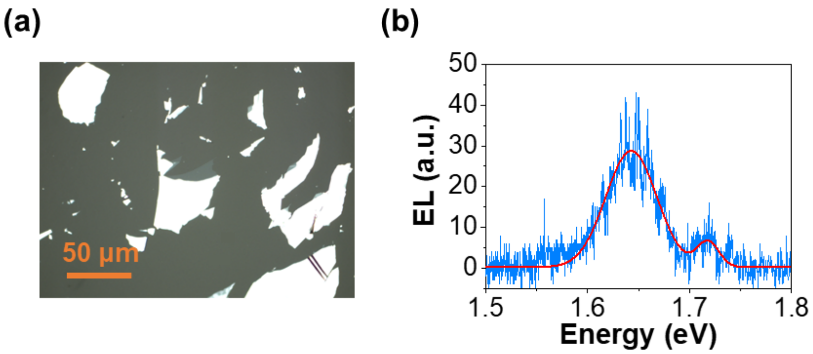

Supporting Information Fig. S1: (**a**) Optical image of monolayer WSe_2 on PDMS. (**b**) The background EL emission from flat WSe_2 region at bias voltage = 3.2 V. There are two peaks, and we attribute the one with lower energy to shallow defect-related localised excitons and the other is from neutral excitons.

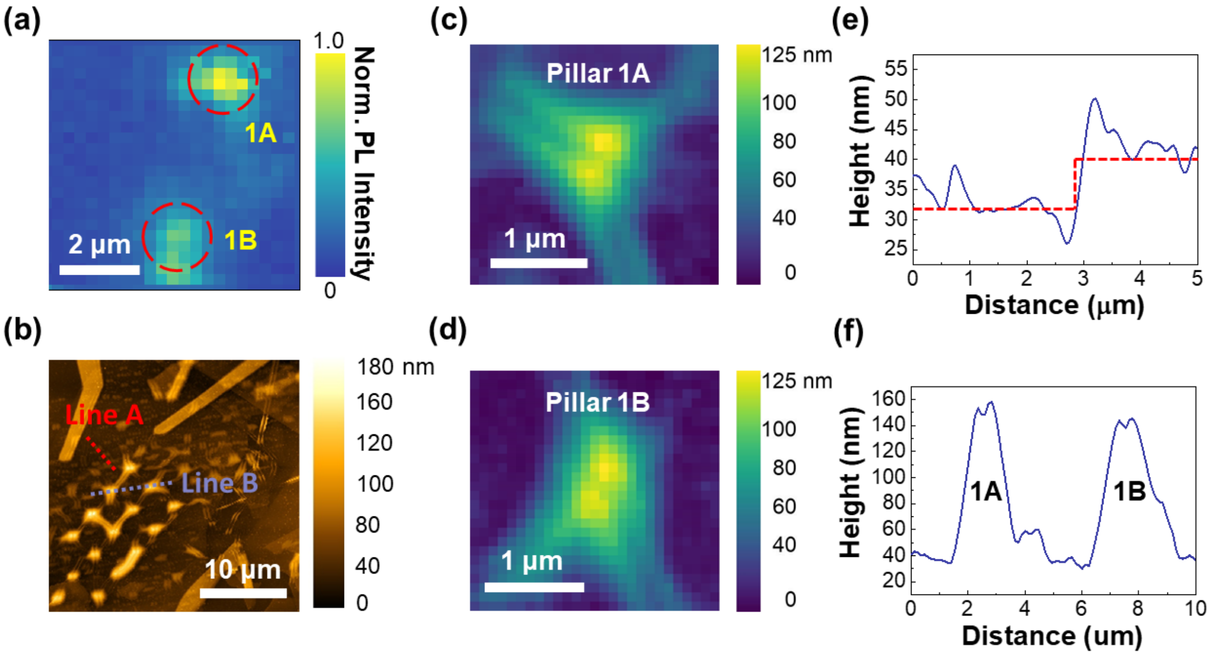

Supporting Information Fig. S2: (**a**) Raster scan of PL intensity over the active region of device 1, with the energy range of 1.5 eV to 1.7 eV. (**b**) AFM measurement of device 1. (**c**) and (**d**) is the high resolution of 3D spacial profile of pillar 1A and pillar 1B. (**e**) is the cross-section height profile of red dashed line A in (**b**) which shows the thickness of the device. (**f**) is the height profile of blue dashed line B in (**b**) which shows the height of the pillar 1A and pillar 1B respectively.

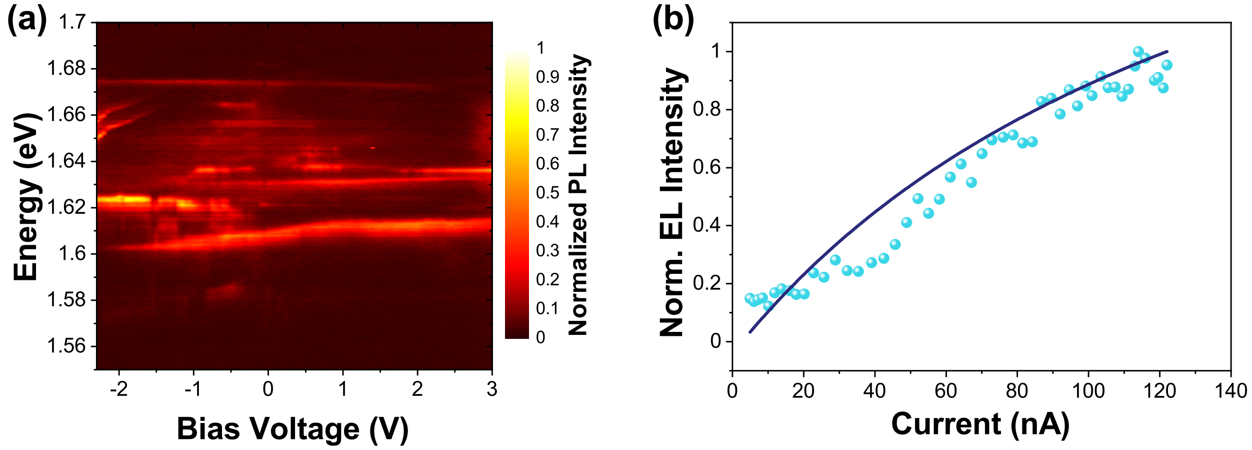

Supporting Information Fig. S3: (**a**) Bias dependent PL spectra from pillar 1A. (**b**) Normalised integrated EL intensity recorded at the pillar 1A as a function of tunnelling current.

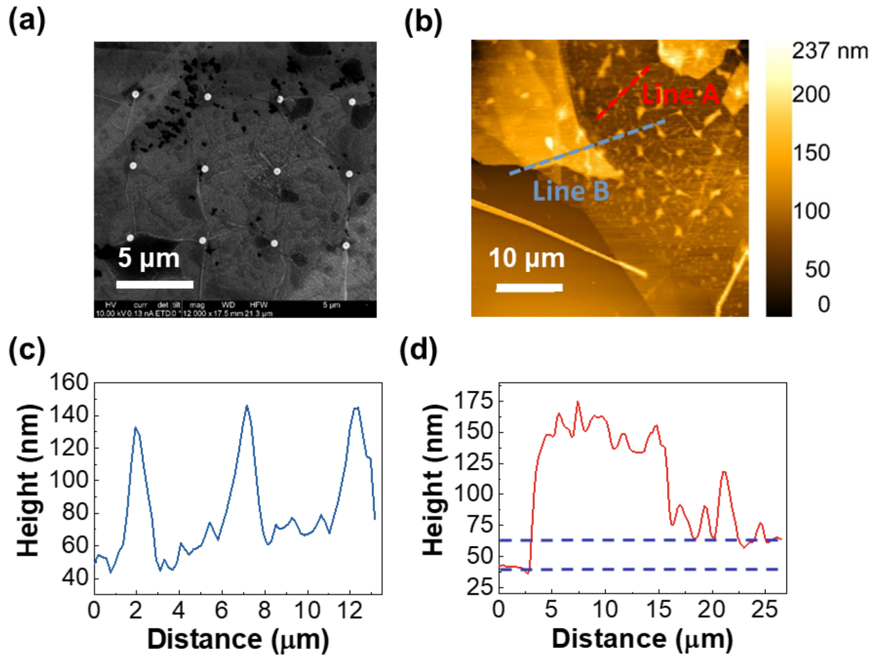

Supporting Information Fig. S4: (**a**) SEM image of pillars in device 2. The scale bar is 5 μm. (**b**) AFM measurement of device 2. The scale bar is 10 μm. (**c**) The cut profile of line A, which shows the height of pillars to be $\sim$ 80 nm. (**d**) The cut profile of line B, which confirms the thickness of device 2 is about 18 nm.

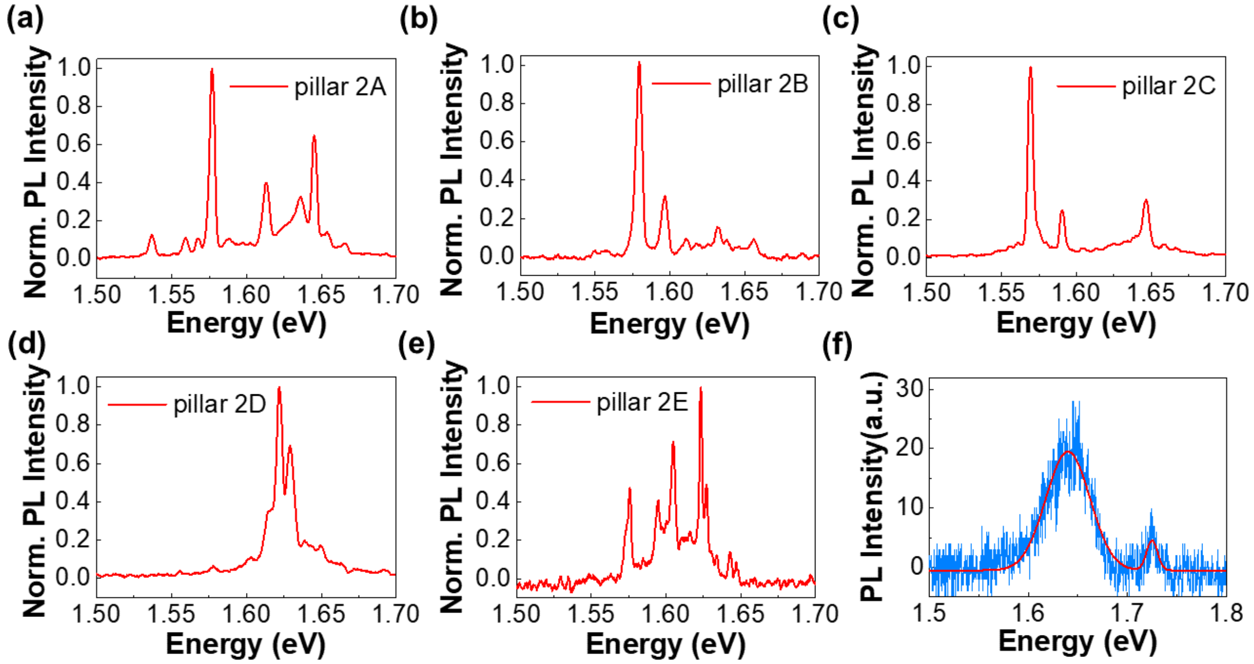

Supporting Information Fig. S5: (**a**) to (**e**) The spectra of PL emission of pillar 2A to 2E without bias. (**f**) The PL spectrum of monolayer WSe_2 from flat region of device 2. The excitation laser is at 650 nm with the power of 2.6 μW/μm^2. Two main peaks are observed. One is stronger emission centered at $\sim$ 1.65eV which can be attributed to shallow-level defects and the other is weaker, from neutral excitons at $\sim$ 1.72eV.

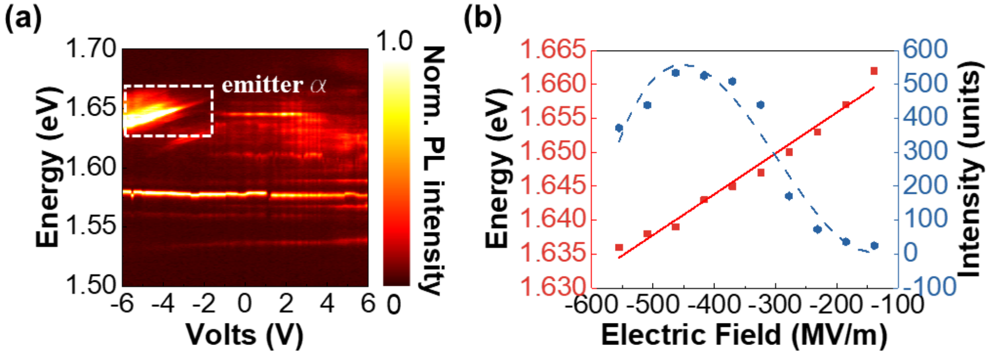

Supporting Information Fig. S6: **PL emission spectra of pillar 2A.** (**a**) The spectral map of pillar 2A as a function of bias. (**b**) The energy and intensity change of emitter-α as a function of electric fields. The dipole moment is calculated to be 2.89 D.

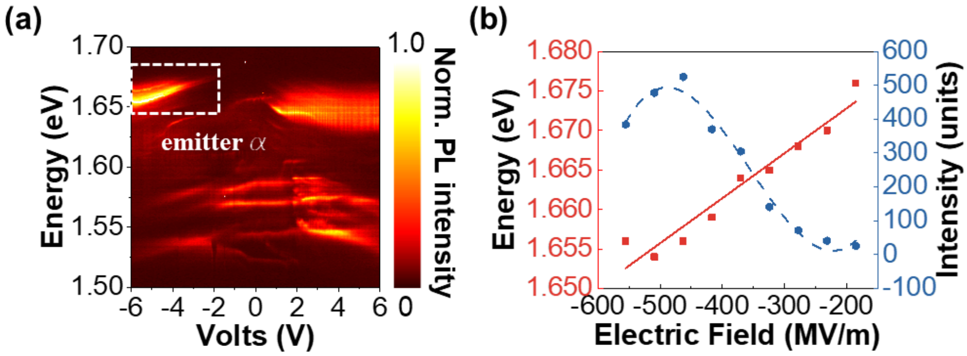

Supporting Information Fig. S7: **PL emission spectra of pillar 2C.** (**a**) The spectral map of pillar 2C as a function of bias. (**b**) The energy and intensity change of emitter-α as a function of electric fields. The dipole moment is calculated to be 2.73 D.

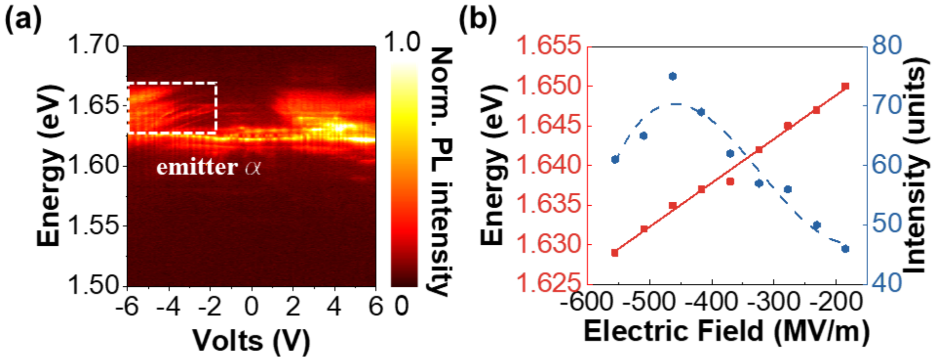

Supporting Information Fig. S8: **PL emission spectra of pillar 2D.** (**a**) The spectral map of pillar 2D as a function of bias. (**b**) The energy and intensity change of emitter-α as a function of electric fields. The dipole moment is calculated to be 2.66 D.

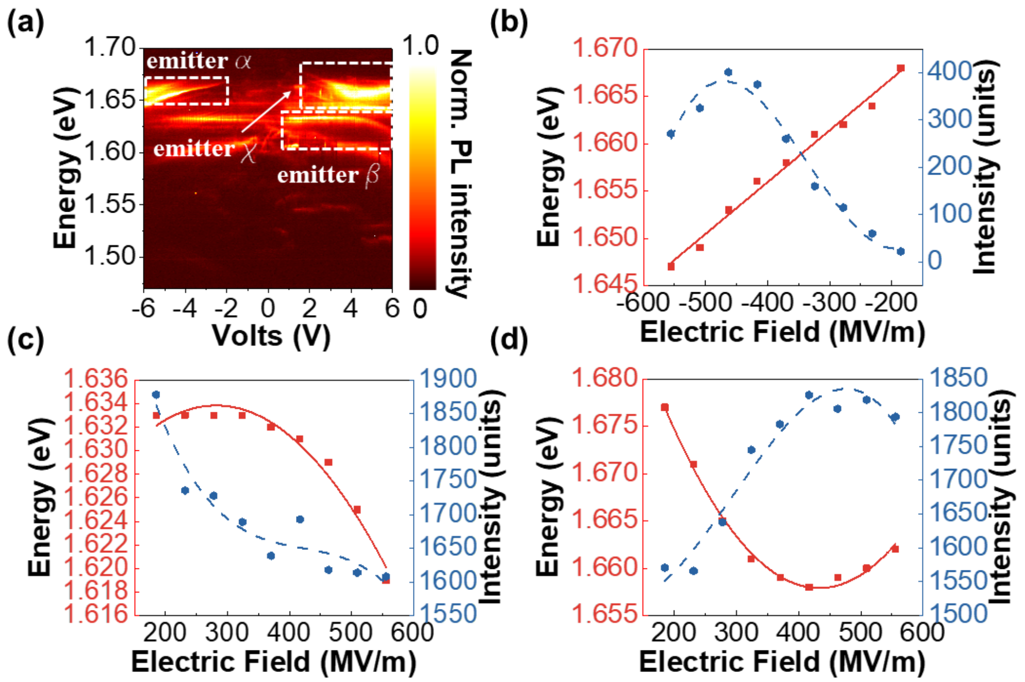

Supporting Information Fig. S9: **PL emission spectra of pillar 2E.** (**a**) The spectral map of pillar 2E as a function of bias. (**b**) to (**d**) The energy and intensity change of emitter-α, emitter-β and emitter-χ as a function of external electric fields respectively. Emitter-α is linear with the dipole moment of 2.62 D. Emitter-β and emitter-χ show quadratic characters with the fitting function to produce the dipole moment of 4.95 D and -3.72 D and polarizability volume of 521 Å and -610 Å respectively.

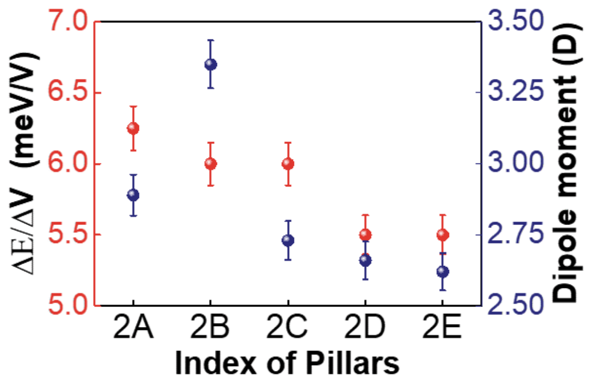

Supporting Information Fig. S10: Change of dipole moment (blue points) and energy change per unit bias (red points) of all 5 pillars in device 2.

References

(1) Withers, F. et al. WSe2 Light-Emitting Tunneling Transistors with Enhanced Brightness at Room Temperature. *Nano Letters* **2015**, *15*, 8223–8228.

(2) Yan, T.; Qiao, X.; Liu, X.; Tan, P.; Zhang, X. Photoluminescence properties and exciton dynamics in monolayer WSe2. *Applied Physics Letters* **2014**, *105*, 101901.

(3) Ye, Y.; Dou, X.; Ding, K.; Chen, Y.; Jiang, D.; Yang, F.; Sun, B. Single photon emission from deep-level defects in monolayer WSe2. *Physical Review B* **2017**, *95*, 245313.

(4) Bishop, S. G.; Hadden, J. P.; Alzahrani, F. D.; Hekmati, R.; Huffaker, D. L.; Langbein, W. W.; Bennett, A. J. Room-Temperature Quantum Emitter in Aluminum Nitride. *ACS Photonics* **2020**, *7*, 1636–1641.
